# Connecting tubules: mechanisms of endoplasmic reticulum membrane fusion

**DOI:** 10.1042/BST20253043

**Published:** 2025-06-30

**Authors:** Eunhong Jang, Youngsoo Jun

**Affiliations:** 1 Department of Life Sciences, Gwangju Institute of Science and Technology, Gwangju 61005, Republic of Korea; 2 Integrated Institute of Biomedical Research, Gwangju Institute of Science and Technology, Gwangju 61005, Republic of Korea

**Keywords:** endoplasmic reticulum, GTPases, membrane fusion, organelle biogenesis

## Abstract

Atlastins (ATLs) are integral dynamin-like GTPases that are critical for the formation and maintenance of the endoplasmic reticulum (ER) network, one of the most complex and essential organelles in eukaryotic cells. The ER, which is composed of interconnected tubules and sheets, serves vital functions, including calcium storage, protein and lipid synthesis, and inter-organelle communication. Homotypic membrane fusion, mediated by ATLs, ensures the tubular structure of the ER by generating and stabilizing three-way junctions. Humans express three ATL paralogs, called ATL1, ATL2, and ATL3, which have distinct expression patterns and regulatory mechanisms. Mutations in these proteins are linked to hereditary sensory neuropathies and hereditary spastic paraplegia, highlighting their critical importance in cellular and neuronal health. Here, we review recent studies providing insights into how ATLs are regulated by their N- and C-terminal extensions, as well as how extrinsic factors potentially regulate the activities of ATLs to establish and maintain the normal ER structure.

The endoplasmic reticulum (ER) is a functionally and morphologically diverse, single-unit, membrane-bound compartment that performs various fundamental cellular processes, including protein translocation and modification, lipid synthesis, and regulation of calcium homeostasis [1]. The ER comprises the nuclear envelope, which is now widely regarded as a specialized subdomain of the ER, and an interconnected network of sheets and tubules, called the peripheral ER. The large surface area of ER sheets provides an optimal site for ribosome binding, thereby enabling protein translocation and folding. The tubular architecture of the ER network permits it to extend and branch throughout the cytoplasm, forming contacts with virtually all other cellular organelles [2–5]. A hallmark of the tubular ER is a distinct structure, termed a three-way junction, which forms upon fusion of the tip of one ER tubule with the side of another ER tubule [6–8]. Atlastins (ATLs), which are integral dynamin-like GTPases, mediate these membrane fusion events between ER tubules through a complex and highly regulated mechanism that relies on their structural organization and GTPase activity [9,10]. Each ATL monomer consists of a dynamin-like GTPase domain, a three-helix bundle (3HB), two transmembrane domains (TMDs), and a cytoplasmic amphipathic helix (Figure 1) [11–13]. These structural components are essential for the stepwise process of membrane tethering and fusion [13–17]. Upon GTP binding, ATL monomers on opposing membranes dimerize via their GTPase domains, initiating a large conformational rearrangement in the adjacent 3HBs to bring membranes into close apposition and promote fusion [16–18]. However, the precise mechanism underlying this fusion process remains controversial, with multiple models proposed based on structural, biochemical, and biophysical studies. Early structural models posited that GTP hydrolysis drives the transition from a prefusion head-to-head dimer to a tightly packed crossover dimer, wherein the 3HBs of each ATL monomer cross-over and interact with the GTPase domain of the opposing partner, which is analogous to a postfusion state [17,19]. However, more recent findings challenge this simple linear pathway. Single-molecule FRET and kinetic analyses suggest that crossover dimerization may occur prior to GTP hydrolysis, which instead facilitates dimer disassembly and ATL recycling, not the fusion event itself [20–22].

**Figure 1:**
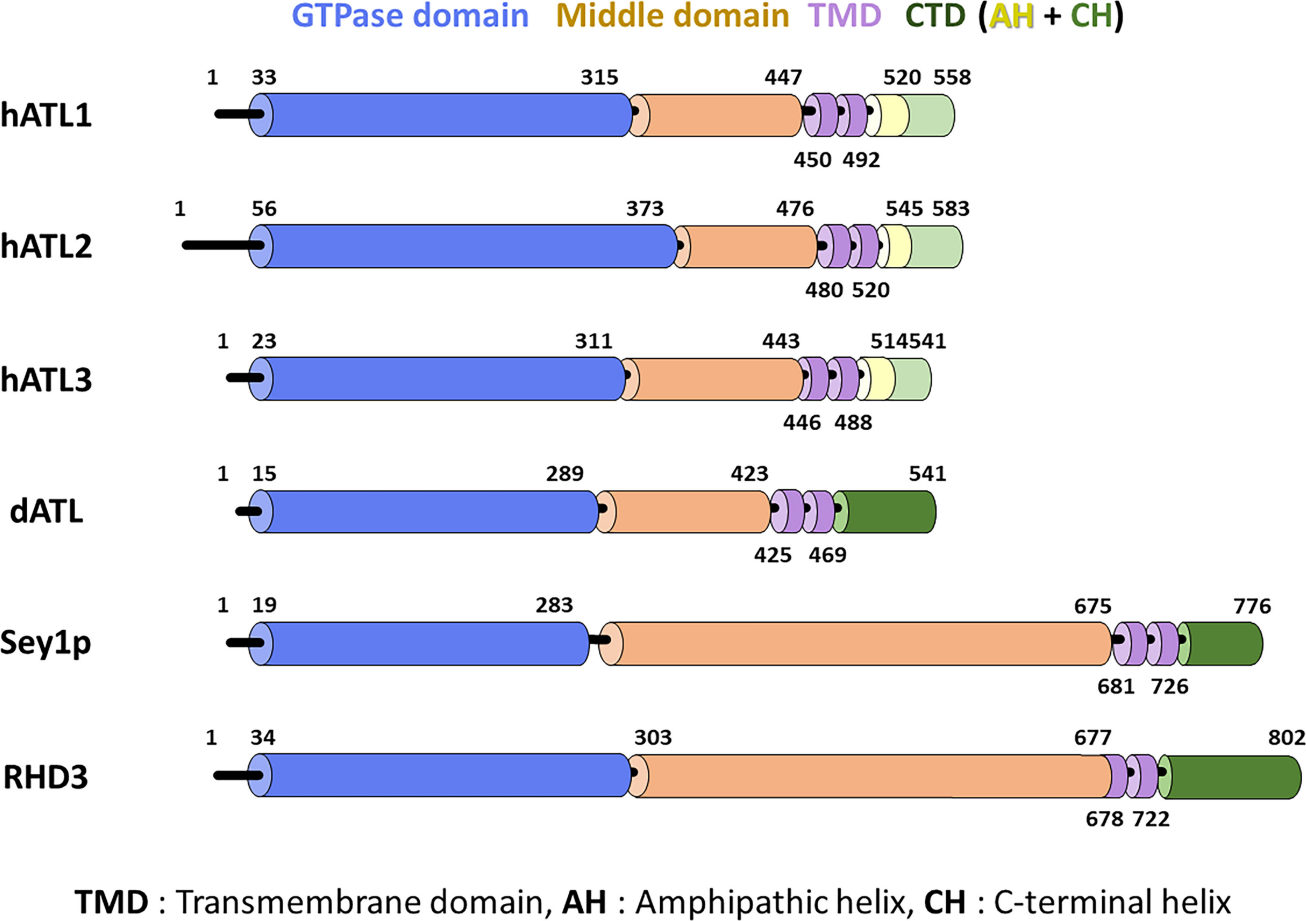
Domain organization of human ATLs and related proteins. Human ATL1, human ATL2, human ATL3, *Drosophila* ATL (dATL), *Saccharomyces cerevisiae* Sey1p, and *Arabidopsis thaliana* RHD3 are shown. The protein diagram shows the number of amino acid residues and where specific domains are located within the structure. While the three human paralogs are organized into five distinct segments (shown as colored cylinders), the remaining proteins are subdivided into four segments. From start to end, the structure consists of an N-terminus of varying length shown in black, a highly preserved GTPase domain shown in blue, a middle domain shown in orange, two consecutive TMDs shown in purple, and a C-terminus of varying length shown in green or a combination of yellow and light green.

In addition to the biochemical steps of fusion, structural details provide critical insights into the mechanistic versatility of ATLs. The amphipathic helix near the C-terminal region plays a pivotal role by partially inserting into and destabilizing the membrane, creating localized curvature that facilitates the fusion process [23]. This is further supported by the unique transmembrane topology of ATLs, which stabilizes the high curvature of the ER network at three-way junctions [24]. Furthermore, crystal structures of ATL homologs reveal conserved dimerization interfaces within the GTPase domain, ensuring precise co-ordination between opposing membranes [16,17,25].

Recent studies have illuminated critical regulatory features unique to human ATLs (Figure 2) [26–29]. Human ATLs exhibit high conservation of their core regions (one dynamin-like GTPase domain, one 3HB, two TMDs, and one cytoplasmic amphipathic helix) but differ in the sequence after their amphipathic helices [C-terminal extension (CTE)] [26–28] and their N-terminal extensions [hypervariable region (HVR)] [29]. According to recent crystal structures of the soluble portions of GDP-bound ATL1 and ATL3, the ATL1 HVR consists of a short β-hairpin, while the ATL3 HVR forms a single α-helix that protrudes from the GTPase domain [29]. Interestingly, the structure suggests that the ATL1 HVR directly contacts the GTPase domain of a neighboring ATL1 molecule in a manner that generates a collection of HVR-dependent oligomers on one membrane. Thus, the ATL1 HVR may co-ordinate the conformational and catalytic cycles of several ATL1 molecules in a membrane. Consistently, by performing a light scattering-based tethering assay, Kelly et al. revealed that deletion of the ATL1 HVR markedly slows tethering [29]. However, the loss of the ATL1 HVR does not affect liposome fusion upon reconstitution into liposomes [26–28]. Thus, the function of the ATL1 HVR remains to be clarified. Furthermore, Jang et al. discovered that the ATL1 HVR contains the binding site for neuronally enriched M1-spastin, which increases the fusion rate of ATL1 upon reconstitution into liposomes together with ATL1 [26]. Thus, the loss of the ATL1 HVR abrogates stimulation by M1-spastin, which otherwise stimulates ATL1-mediated liposome fusion [26].

**Figure 2:**
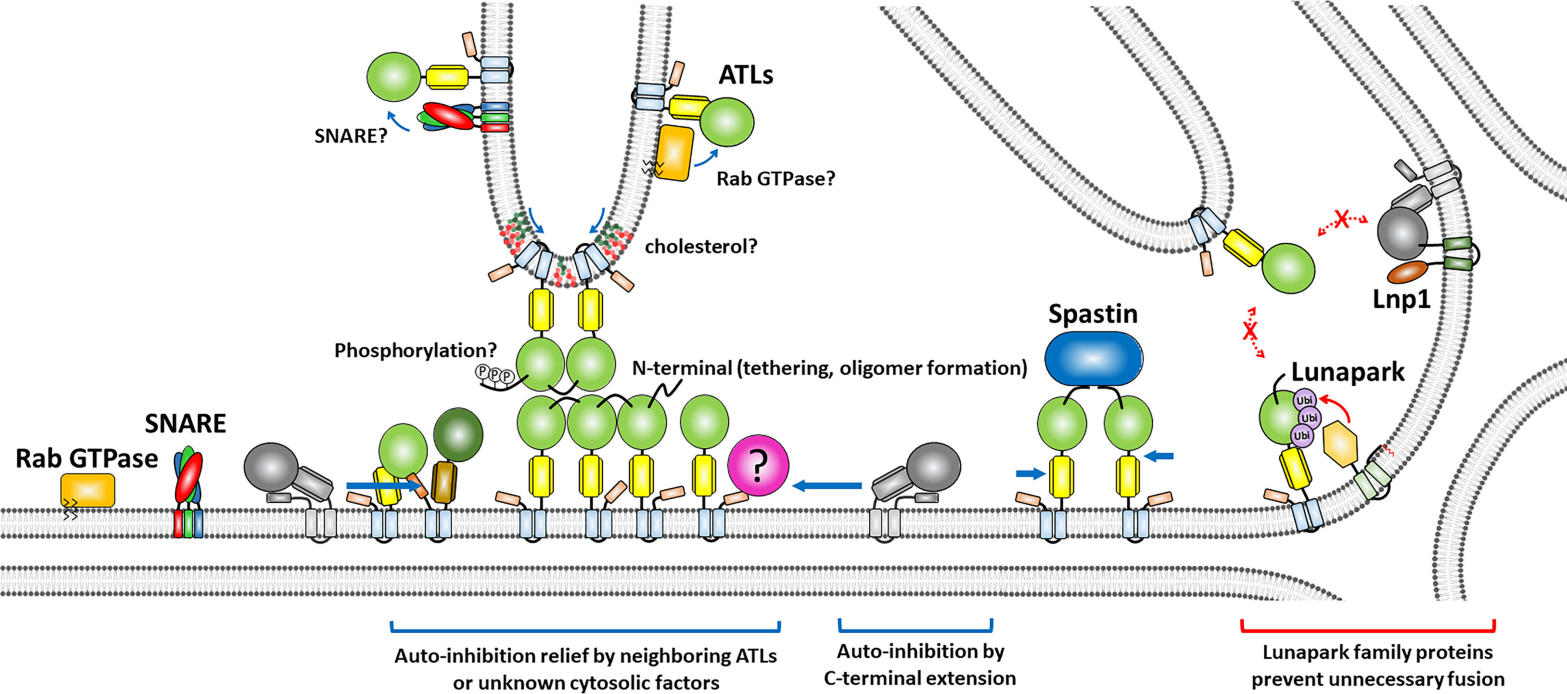
Intrinsic and extrinsic modes of ATL regulation.

According to the study by Kelly et al., the ATL3 HVR forms an α-helix that protrudes from the GTPase domain and possibly contacts the GTPase domain of a neighboring ATL3 molecule [29]. Despite this, deletion of the ATL3 HVR does not compromise the membrane tethering kinetics of ATL3 [29]. Interestingly, however, deletion of the HVR (Δ1–18) almost completely abolishes ATL3-mediated liposome fusion [26], reflecting the functional importance of the ATL3 HVR for ATL3-mediated fusion. These results collectively suggest that the ATL3 HVR plays a role after the membrane tethering step but before the lipid mixing step during ATL3-mediated membrane fusion.

Unlike the HVRs of ATL1 and ATL3, it remains unknown whether the ATL2 HVR has a role in membrane tethering or fusion. Although ATL2 structures have not been reported, the AlphaFold prediction of ATL2 suggests that its HVR is largely disordered. Thus, it would be interesting to determine whether the disordered HVR of ATL2 has any role in ATL2-mediated ER membrane fusion.

The CTE is alternatively spliced in ATL1 and ATL2. Two ATL1 variants (ATL1-1 and ATL1-2) and three ATL2 variants (ATL2-1, ATL2-2, and ATL2-3) have been biochemically characterized [26–28]. Deletion of the CTE from ATL1-1 and ATL1-2 markedly increases fusion activity [27]. The difference between ATL1-1 and ATL1-2 is that the former contains an additional five amino acids (GSTNE). Interestingly, C-terminal autoinhibition, which is observed in ATL1-1, is weakened by the absence of the GSTNE motif in ATL1-2 [27].

ATL2-1, which is the major splicing variant of ATL2, has almost no fusion activity upon reconstitution into liposomes, but an ATL2 mutant construct lacking the ATL2-1 CTE exhibits strong fusion activity [26–28]. By contrast, the CTEs of ATL2-2 and ATL2-3 have almost no or little inhibitory activity [26–28]. Unlike the splicing variants of ATL1, the absence of the RSPRK motif, which is found in ATL2-3, modestly strengthens C-terminal autoinhibition in ATL2-2 [27]. ATL3 has fusion activity on its own, which is unaffected by the removal of its CTE, indicating that ATL3 is a constitutive fusion catalyst [27]. Therefore, human cells possess several options to regulate ER fusion.

Although ATL2-1 has nearly no fusion activity due to C-terminal autoinhibition when reconstituted into liposomes, it seems to readily become active *in vivo* [26]. ATL2-1 is the predominant ATL in HEK293 cells, and efficient fusion of HEK293 cell-derived ER microsomes is heavily dependent on ATL2-1 because this fusion is completely blocked in the presence of an anti-ATL2-1 antibody [26]. Although it remains unknown how the autoinhibition is relieved *in vivo*, our study proposed two potential scenarios [26]. First, ATL3, although present in small quantities in HEK293 cells, may relieve the autoinhibition of ATL2-1 by interacting with its CTE, and thereby preventing it from binding to the GTPase domain of ATL2-1. Second, a cytoplasmic factor may bind to ATL2-1 and thereby relieve the autoinhibition because liposomes bearing ATL2-1 became fusogenic in the presence of isolated cytosol.

Thus, the three human ATL paralogs exhibit significant functional diversity, reflecting their tissue-specific roles and regulatory mechanisms. ATL1 is predominantly expressed in neurons, while ATL2 and ATL3 are expressed more ubiquitously in tissues. ATL3 operates as a constitutive ER fusion catalyst, lacking the autoinhibition observed in ATL1 and ATL2. These differences suggest that evolutionary adaptations occurred to meet the unique demands of different cell types. For example, a neuronal-specific splice variant of ATL2 (ATL2-2) exhibits higher fusion activity than a ubiquitously expressed isoform (ATL2-1), demonstrating how splicing events can adapt ATL functionality to specific cellular contexts [26–28].

Extrinsic regulatory mechanisms may further modulate ATL activity. The *in vitro* fusion activities of bacterially expressed human ATLs were not observed until recently; therefore, post-translational modifications, such as phosphorylation, were suspected to be required for the fusion activities of human ATLs. When ATL1 was purified from a HEK293 cell-derived suspension cell line, S22 and S23 of ATL1 were found to be heavily phosphorylated [28]. However, fusion activity was not due to this phosphorylation because it was unaffected by alanine substitution of both sites [28]. Kelly et al. found additional phosphorylation at S10 of ATL1 [29]. In their study, mutations at these three serine residues (S10, S22, and S23) differentially affected membrane tethering and ER morphology [29]. However, Jang et al. successfully reconstituted the fusion activities of all three human ATLs using bacterially expressed ATLs [26]; therefore, post-translational modifications may not be essential for the fusion activities of human ATLs *per se*. Nonetheless, external signaling pathways may fine-tune ATL activity in response to cellular needs.

Recent advances in the study of ATLs have highlighted the role of lipid composition in modulating their fusion activities [26,30,31]. Physiological lipid mixtures that mimic the ER membrane enhance ATL-mediated fusion, suggesting that the lipid environment has an important role in regulating ATL function [26]. This finding provides new avenues to explore how membrane composition influences ER dynamics. Proteins function as catalysts during membrane fusion in general, whereas lipids were long thought to only have structural functions. However, emerging evidence suggests that lipids also directly regulate membrane fusion, such as SNARE-mediated and viral protein-mediated membrane fusion [32–35]. Such lipids, including phosphoinositides, sterols, diacylglycerol, and phosphatidic acid, are called ‘regulatory lipids’ because they have more functional than structural roles in membrane fusion [36]. Additionally, the inverted cone-shaped molecular structure of phosphatidylethanolamine (PE) induces negative membrane curvature and assists the hemifusion structure, where two lipid bilayers partially merge, allowing the outer leaflets to mix, while the inner leaflets and aqueous contents remain distinct, to catalyze fusion [37]. Cholesterol has intrinsic negative curvature and thus may reduce the amount of energy required to form lipid stalks in hemifusion intermediates and stabilize fusion pores. It might also directly control the stability and formation of fusion pores [33]. The ER also contains these regulatory lipids; therefore, we used a lipid mixture, whose composition resembles that of the ER, to reconstitute human ATLs into liposomes and successfully observed fusion activity [26]. Omission of cholesterol alone or cholesterol plus PE from the physiological lipid mixture markedly reduces ATL2-mediated fusion [26]. Nonetheless, these regulatory lipids may not be essential for human ATL-mediated fusion *per se* because the Lee laboratory did not include any of these regulatory lipids to observe human ATL-mediated liposome fusion when ATLs were purified from cultured human cells [27,28].

Furthermore, a study by Joji Mima’s laboratory revealed that Sey1p, the yeast ortholog of human ATLs, requires regulatory lipids, including ergosterol (yeast cholesterol), phosphatidylinositol, and phosphatidic acid, for efficient fusion [30]. Our laboratory also demonstrated that Sey1p harbors two sterol-binding motifs close to its TMDs. Disruption of these motifs markedly abrogates the interaction of Sey1p with sterols and markedly decreases Sey1p-mediated ER fusion [31]. Interestingly, each human ATL contains potential sterol-binding motifs near its TMDs [31]. Thus, it would be intriguing to investigate whether cholesterol also interacts with human ATLs through these motifs and, thus, enhances human ATL-mediated ER membrane fusion.

The pathological significance of human ATLs is most evident in hereditary spastic paraplegia (HSP) and hereditary sensory neuropathies (HSNs) [38]. Mutations in ATL1 are a leading cause of HSP, which is a neurodegenerative condition characterized by spasticity in the lower limbs and progressive weakness [39]. Similarly, mutations in ATL3 are implicated in HSNs [40,41]. The fusion activities of human ATLs were successfully reconstituted *in vitro* recently, and thus, how disease-causing mutations affect the fusion activities of ATLs can now be analyzed. While many ATL-associated, disease-causing mutations disrupt ER network formation by impairing GTP hydrolysis, crossover dimerization, or membrane fusion [16,17], several clinically relevant variants exhibit near wildtype fusion activity *in vitro*, but still cause HSP [23]. For example, the SPG3A-linked mutations R239C and H258R elicit opposite effects on liposome fusion, namely, reduction and enhancement, respectively, but both cause disease, suggesting that fusion efficiency alone does not determine pathogenicity [42]. Likewise, some ATL1 mutations do not significantly impair fusion or ER morphology but still induce axon growth defects in neuronal models [43]. By contrast, ATL3 with the HSN-causing mutations Y192C and P338R has no observable fusion activity [26,27]. These observations point to fusion-independent mechanisms, including misregulated ER–microtubule interactions (via spastin or REEP1), disturbed ER–mitochondria coupling, or altered protein translation and autophagy, particularly in long motor neurons [44,45]. Therefore, ATL mutations may contribute to disease through diverse, context-dependent pathways, only some of which involve membrane fusion *per se*.

A recent study reported that the expression of ATL2-2, an uninhibited splicing variant, is elevated in breast tumors compared with normal breast tissue. Furthermore, high ATL2-2 mRNA expression is associated with basal-like, estrogen receptor-negative, large, and high-grade tumors, which are all indicative of a worse prognosis [46]. These results suggest that tight regulation of the fusion activity of ATL2 is critical for normal cell proliferation.

Comparative studies of ATLs and their orthologs, such as Root Hair Defective 3 (RHD3) in plants [47] and Sey1p in yeast [31,48,49], have provided valuable evolutionary insights. Unlike mammalian ATLs, these orthologs exhibit robust fusion activities without autoinhibition by the CTE, reflecting the simpler regulatory demands of unicellular and plant systems [50]. These comparisons highlight the evolutionary pressures that have shaped ATL function to meet the complex needs of multicellular organisms. Our laboratory has demonstrated that ER fusion requires not only Sey1p but also ER-resident SNARE proteins in the budding yeast *Saccharomyces cerevisiae* [31]. This was a surprising finding because a single fusion event involves two distinct fusion machineries: the ATL-like GTPase Sey1p and SNAREs, which are usually involved in the fusion of transport vesicles with target organelles [51]. Interestingly, however, Rab GTPases, which are often essential for SNARE-mediated fusion [52], are not required for yeast ER fusion [31]. There is accumulating evidence that ER-resident Rab GTPases [53,54] and SNAREs [55] play a role in regulating the human ER structure; therefore, it would be intriguing to examine whether human SNAREs and Rab GTPases are involved in ATL-mediated ER membrane fusion. In plants, the ATL homolog RHD3 plays an essential role in ER morphogenesis and is particularly required for the development of fine polarized structures such as root hairs. Loss of RHD3 function results in shortened root hairs and disrupted ER networks, which are reminiscent of the defects in axonal development observed upon loss of mammalian ATL1 [47,56,57]. This conservation across species underscores the fundamental role of ATL-like GTPases in shaping ER structure to support highly elongated cellular processes.

Another emerging area of research involves the interactions of ATLs with other ER-shaping proteins. For instance, reticulons (RTNs) and REEP proteins are curvature-stabilizing proteins that insert into the membrane via hydrophobic hairpin domains and generate high membrane curvature, essential for tubule formation [58–60]. RTNs can also oppose ATL function by promoting membrane constriction and fission, as shown in recent *in vitro* and *in vivo* studies. In *Drosophila*, for example, ER fragmentation caused by ATL loss is rescued by concurrent deletion of RTN, indicating that there is an antagonistic balance between fusion and fission [61]. M1-spastin, a microtubule-severing ATPase, further contributes to ER morphology by interacting with ATL1 and REEP1, thereby linking ER tubules to the microtubule cytoskeleton and co-ordinating their extension and distribution [62]. These interactions are especially critical in neurons, where long axons demand precise ER architecture for intracellular transport. Notably, mutations in ATL1, M1-spastin (SPG4), and REEP1 (SPG31) are among the most common genetic causes of HSP, highlighting their functional interdependence in disease contexts [6].

Among the proteins that interact with ATLs, lunapark may be the only one that antagonizes their functions; however, its mode of action is largely not known [63]. Lunapark is also reportedly involved in the stabilization of three-way junctions [64]. Consequently, lunapark may fine-tune the ER structure by preventing excessive three-way junction formation and stabilizing preformed three-way junctions upon alterations of the intracellular environment. Lunapark is an E3 ligase for ATLs, which is one of its most well-defined functions [65]. For instance, the N-terminal cytoplasmic domain of human lunapark possesses ubiquitin ligase activity to ubiquitinylate ATL2 [66]. In our recent study [67], we demonstrated that the yeast lunapark Lnp1p prevents Sey1p-mediated ER fusion by inhibiting trans-Sey1p complex formation, which is a prerequisite for ER membrane tethering and fusion, via its interaction with the GTPase domain of Sey1p. Unlike human lunapark, Lnp1p does not seem to have E3 ligase activity because its deletion does not affect the steady-state level of Sey1p. Considering that the fusion activities of all human ATLs have been successfully reconstituted *in vitro* [26,28], it would be intriguing to examine whether human lunapark negatively regulates human ATL-mediated fusion similarly to yeast lunapark.

## Conclusions and future outlook

ER membrane fusion is a highly co-ordinated process that ensures dynamic remodeling and maintenance of the ER network. Among the molecular players that mediate this process, ATLs function as the core machinery of ER tubule fusion, with their mechanistic versatility underscoring the complexity of ER dynamics. The intricate interplay of structural domains within ATLs, including their GTPase domain, amphipathic helix, and TMDs, facilitates efficient tethering, fusion, and remodeling of ER membranes. Recent structural and biochemical studies have revealed novel regulatory elements, such as the HVR and CTE, which fine-tune the functions of ATLs in a paralog-specific and tissue-dependent manner [26–29]. These findings highlight the evolutionary adaptations of ATLs to diverse cellular environments, providing an additional layer of complexity in ER network regulation.

Beyond intrinsic structural elements, extrinsic factors also shape the functions of ATLs. Post-translational modifications, lipid composition, and interactions with ER-shaping proteins such as spastin and lunapark further modulate the efficiency of ER fusion. Notably, the emerging involvement of regulatory lipids in ATL-mediated fusion challenges the traditional view of membrane lipids as passive structural components and instead positions them as active participants in ER dynamics.

Future research must address several open questions, including the mechanisms underlying relief of ATL autoinhibition *in vivo*, the role of sterol-binding motifs in human ATLs, and the potential involvement of Rab GTPases and SNAREs in ATL-mediated ER fusion. Furthermore, elucidation of the antagonistic role of lunapark in the functions of ATLs may provide new insights into how ER homeostasis is maintained under physiological and pathological conditions.

Overall, the study of ER membrane fusion has reached an exciting juncture, with advances in structural biology and *in vitro* reconstitution assays providing unprecedented mechanistic clarity. Continued exploration of ATLs and their regulatory networks will deepen our understanding of ER dynamics and help to develop novel therapeutic strategies for ER-associated diseases.

**Perspectives**

- The dynamic network structure of endoplasmic reticulum (ER), which is maintained through regulated membrane fusion events, is fundamental to cellular homeostasis and inter-organelle communication, and its disruption is linked to severe neurological disorders, including hereditary spastic paraplegia and hereditary sensory neuropathies.
- Recent structural and biochemical studies have revealed that human atlastins (ATLs) employ sophisticated regulatory mechanisms through their N- and C-terminal extensions, with distinct tissue-specific expression patterns and varying degrees of autoinhibition, while regulatory lipids and protein partners provide additional layers of control over ER membrane fusion.
- Future research should focus on understanding how atlastin autoinhibition is relieved *in vivo*, exploring the potential roles of sterol-binding motifs in human ATLs, and investigating whether ER-resident SNAREs and Rab GTPases co-operate with ATLs in membrane fusion, as observed in simpler organisms.

## Open Access

This article has been published open access under our Subscribe to Open programme, made possible through the support of our subscribing institutions, learn more here: https://portlandpress.com/pages/open_access_options_and_prices#conditional

## Abbreviations

ATLs: atlastins
CTE: C-terminal extension
ER: endoplasmic reticulum
3HB: three-helix bundle
HSNs: hereditary sensory neuropathies
HSP: hereditary spastic paraplegia
HVR: hypervariable region
PE: phosphatidylethanolamine
REEP: Receptor Expression-Enhancing Protein
RHD3: Root Hair Defective 3
RTNs: reticulons
SNARE: Soluble N-ethylmaleimide-sensitive factor Attachment protein REceptor
TMDs: transmembrane domains

## References

[1] Westrate L.M. Lee J.E. Prinz W.A. Voeltz G.K 2015 Form follows function: the importance of endoplasmic reticulum shape Annu. Rev. Biochem. 84 791 811 10.1146/annurev-biochem-072711-163501 25580528

[2] Chen S. Novick P. Ferro-Novick S 2013 ER structure and function Curr. Opin. Cell Biol 25 428 433 10.1016/j.ceb.2013.02.006 23478217 PMC5614462

[3] Friedman J.R. Voeltz G.K 2011 The ER in 3D: a multifunctional dynamic membrane network Trends Cell Biol. 21 709 717 10.1016/j.tcb.2011.07.004 21900009 PMC3221873

[4] English A.R. Zurek N. Voeltz G.K 2009 Peripheral ER structure and function Curr. Opin. Cell Biol. 21 596 602 10.1016/j.ceb.2009.04.004 19447593 PMC2753178

[5] Estrada de Martin P. Novick P. Ferro-Novick S 2005 The organization, structure, and inheritance of the ER in higher and lower eukaryotes Biochem. Cell Biol. 83 752 761 10.1139/o05-159 16333327

[6] Goyal U. Blackstone C 2013 Untangling the web: Mechanisms underlying ER network formation Biochimica et Biophysica Acta (BBA) - Molecular Cell Research 1833 2492 2498 10.1016/j.bbamcr.2013.04.009 23602970 PMC3729797

[7] Pendin D. McNew J.A. Daga A 2011 Balancing ER dynamics: shaping, bending, severing, and mending membranes Curr. Opin. Cell Biol. 23 435 442 10.1016/j.ceb.2011.04.007 21641197 PMC3148315

[8] Moss T.J. Daga A. McNew J.A 2011 Fusing a lasting relationship between ER tubules Trends Cell Biol. 21 416 423 10.1016/j.tcb.2011.03.009 21550242 PMC3128651

[9] Orso G. Pendin D. Liu S. Tosetto J. Moss T.J. Faust J.E. et al 2009 Homotypic fusion of ER membranes requires the dynamin-like GTPase atlastin Nature New Biol. 460 978 983 10.1038/nature08280 19633650

[10] Hu J. Shibata Y. Zhu P.-P. Voss C. Rismanchi N. Prinz W.A. et al 2009 A class of dynamin-like GTPases involved in the generation of the tubular ER network Cell 138 549 561 10.1016/j.cell.2009.05.025 19665976 PMC2746359

[11] Hu J. Rapoport T.A 2016 Fusion of the endoplasmic reticulum by membrane-bound GTPases Semin. Cell Dev. Biol. 60 105 111 10.1016/j.semcdb.2016.06.001 27269373

[12] McNew J.A. Sondermann H. Lee T. Stern M. Brandizzi F 2013 GTP-dependent membrane fusion Annu. Rev. Cell Dev. Biol. 29 529 550 10.1146/annurev-cellbio-101512-122328 23875647

[13] Moss T.J. Andreazza C. Verma A. Daga A. McNew J.A 2011 Membrane fusion by the GTPase atlastin requires a conserved C-terminal cytoplasmic tail and dimerization through the middle domain Proc. Natl. Acad. Sci. U.S.A. 108 11133 11138 10.1073/pnas.1105056108 21690399 PMC3131361

[14] Shi L. Yang C. Zhang M. Li K. Wang K. Jiao L. et al 2024 Dissecting the mechanism of atlastin-mediated homotypic membrane fusion at the single-molecule level Nat. Commun. 15 2488. 10.1038/s41467-024-46919-z 38509071 PMC10954664

[15] O’Donnell J.P. Cooley R.B. Kelly C.M. Miller K. Andersen O.S. Rusinova R . et al 2017 Timing and reset mechanism of GTP hydrolysis-driven conformational changes of atlastin Structure 25 997 1010 10.1016/j.str.2017.05.007 28602821 PMC5516944

[16] Byrnes L.J. Sondermann H 2011 Structural basis for the nucleotide-dependent dimerization of the large G protein atlastin-1/SPG3A Proc. Natl. Acad. Sci. U.S.A. 108 2216 2221 10.1073/pnas.1012792108 21220294 PMC3038741

[17] Bian X. Klemm R.W. Liu T.Y. Zhang M. Sun S. Sui X. et al 2011 Structures of the atlastin GTPase provide insight into homotypic fusion of endoplasmic reticulum membranes Proc. Natl. Acad. Sci. U.S.A. 108 3976 3981 10.1073/pnas.1101643108 21368113 PMC3054032

[18] Liu T.Y. Bian X. Sun S. Hu X. Klemm R.W. Prinz W.A. et al 2012 Lipid interaction of the C terminus and association of the transmembrane segments facilitate atlastin-mediated homotypic endoplasmic reticulum fusion Proc. Natl. Acad. Sci. U.S.A. 109 E2146 54 10.1073/pnas.1208385109 22802620 PMC3420179

[19] Byrnes L.J. Singh A. Szeto K. Benvin N.M. O’Donnell J.P. Zipfel W.R. et al 2013 Structural basis for conformational switching and GTP loading of the large G protein atlastin EMBO J. 32 369 384 10.1038/emboj.2012.353 23334294 PMC3567502

[20] Crosby D. Lee T.H 2022 Membrane fusion by *Drosophila* atlastin does not require GTP hydrolysis Mol. Biol. Cell 33 br23 10.1091/mbc.E22-05-0164 36129776 PMC9727788

[21] Winsor J. Machi U. Han Q. Hackney D.D. Lee T.H 2018 GTP hydrolysis promotes disassembly of the atlastin crossover dimer during ER fusion J. Cell Biol. 217 4184 4198 10.1083/jcb.201805039 30249723 PMC6279388

[22] Winsor J. Hackney D.D. Lee T.H 2017 The crossover conformational shift of the GTPase atlastin provides the energy driving ER fusion J. Cell Biol. 216 1321 1335 10.1083/jcb.201609071 28356327 PMC5412568

[23] Faust J.E. Desai T. Verma A. Ulengin I. Sun T.-L. Moss T.J. et al 2015 The Atlastin C-terminal tail is an amphipathic helix that perturbs the bilayer structure during endoplasmic reticulum homotypic fusion J. Biol. Chem. 290 4772 4783, S0021-9258(19)46884-1 10.1074/jbc.M114.601823 25555915 PMC4335215

[24] Betancourt-Solis M.A. Desai T. McNew J.A 2018 The atlastin membrane anchor forms an intramembrane hairpin that does not span the phospholipid bilayer Journal of Biological Chemistry 293 18514 18524 10.1074/jbc.RA118.003812 30287684 PMC6290144

[25] Liu T.Y. Bian X. Romano F.B. Shemesh T. Rapoport T.A. Hu J 2015 Cis and trans interactions between atlastin molecules during membrane fusion Proc. Natl. Acad. Sci. U.S.A. 112 E1851 60 10.1073/pnas.1504368112 25825753 PMC4403200

[26] Jang E. Moon Y. Yoon S.Y. Diaz J.A.R. Lee M. Ko N. et al 2023 Human atlastins are sufficient to drive the fusion of liposomes with a physiological lipid composition J. Cell Biol. 222 e202109090 10.1083/jcb.202109090 36757370 PMC9949273

[27] Bryce S. Stolzer M. Crosby D. Yang R. Durand D. Lee T.H 2023 Human atlastin-3 is a constitutive ER membrane fusion catalyst J. Cell Biol. 222 e202211021 10.1083/jcb.202211021 37102997 PMC10140384

[28] Crosby D. Mikolaj M.R. Nyenhuis S.B. Bryce S. Hinshaw J.E. Lee T.H 2022 Reconstitution of human atlastin fusion activity reveals autoinhibition by the C terminus J. Cell Biol. 221 e202107070 10.1083/jcb.202107070 34817557 PMC8624677

[29] Kelly C.M. Byrnes L.J. Neela N. Sondermann H. and O’Donnell J.P 2021 The hypervariable region of atlastin-1 is a site for intrinsic and extrinsic regulation J. Cell Biol. 220 e202104128 10.1083/jcb.202104128 34546351 PMC8563291

[30] Sugiura S. Mima J 2016 Physiological lipid composition is vital for homotypic ER membrane fusion mediated by the dynamin-related GTPase Sey1p Sci. Rep. 6 20407 10.1038/srep20407 26838333 PMC4738300

[31] Lee M. Ko Y.-J. Moon Y. Han M. Kim H.-W. Lee S.H. et al 2015 SNAREs support atlastin-mediated homotypic ER fusion in Saccharomyces cerevisiae J. Cell Biol. 210 451 470 10.1083/jcb.201501043 26216899 PMC4523606

[32] Jahn R. Cafiso D.C. Tamm L.K 2024 Mechanisms of SNARE proteins in membrane fusion Nat. Rev. Mol. Cell Biol. 25 101 118 10.1038/s41580-023-00668-x 37848589 PMC11578640

[33] Yang S.-T. Kreutzberger A.J.B. Lee J. Kiessling V. Tamm L.K 2016 The role of cholesterol in membrane fusion Chem. Phys. Lipids 199 136 143 10.1016/j.chemphyslip.2016.05.003 27179407 PMC4972649

[34] Kozlov M.M. Chernomordik L.V 2015 Membrane tension and membrane fusion Curr. Opin. Struct. Biol. 33 61 67 10.1016/j.sbi.2015.07.010 26282924 PMC4641764

[35] Wickner W 2010 Membrane fusion: five lipids, four SNAREs, three chaperones, two nucleotides, and a Rab, all dancing in a ring on yeast vacuoles Annu. Rev. Cell Dev. Biol. 26 115 136 10.1146/annurev-cellbio-100109-104131 20521906

[36] Fratti R.A. Jun Y. Merz A.J. Margolis N. Wickner W 2004 Interdependent assembly of specific regulatory lipids and membrane fusion proteins into the vertex ring domain of docked vacuoles J. Cell Biol. 167 1087 1098 10.1083/jcb.200409068 15611334 PMC2172599

[37] Sardar A. Dewangan N. Panda B. Bhowmick D. Tarafdar P.K 2022 Lipid and Lipidation in Membrane Fusion J. Membr. Biol. 255 691 703 10.1007/s00232-022-00267-5 36102950 PMC9472184

[38] Sonda S. Pendin D. Daga A 2021 ER Morphology in the pathogenesis of hereditary spastic paraplegia Cells 10 2870 10.3390/cells10112870 34831093 PMC8616106

[39] Zhao X. Alvarado D. Rainier S. Lemons R. Hedera P. Weber C.H. et al 2001 Mutations in a newly identified GTPase gene cause autosomal dominant hereditary spastic paraplegia Nat. Genet. 29 326 331 10.1038/ng758 11685207

[40] Mohammadi S. Jafari Khamirani H. Baneshi M. Kamal N. Manoocheri J. Saffar M. et al 2023 A novel nonsense variant in the ATL3 gene is associated with disturbed pain sensitivity, numbness of distal limbs and muscle weakness Ann. Hum. Genet. 87 147 157 10.1111/ahg.12501 36856139

[41] Fischer D. Schabhüttl M. Wieland T. Windhager R. Strom T.M. Auer-Grumbach M 2014 A novel missense mutation confirms ATL3 as a gene for hereditary sensory neuropathy type 1 Brain (Bacau) 137 e286 e286, e286 10.1093/brain/awu091 24736309

[42] Hocquel A. Ravel J.-M. Lambert L. Bonnet C. Banneau G. Kol B. et al 2022 Reduced penetrance of an eastern French mutation in ATL1 autosomal-dominant inheritance (SPG3A): extended phenotypic spectrum coupled with brain ^18^F-FDG PET Neurogenetics 23 241 255 10.1007/s10048-022-00695-4 35788923

[43] Zhu P.-P. Denton K.R. Pierson T.M. Li X.-J. Blackstone C 2014 Pharmacologic rescue of axon growth defects in a human iPSC model of hereditary spastic paraplegia SPG3A Hum. Mol. Genet. 23 5638 5648 10.1093/hmg/ddu280 24908668 PMC4189900

[44] Lu X. Yang M. Yang Y. Wang X 2020 Atlastin‐1 modulates seizure activity and neuronal excitability CNS Neurosci. Ther. 26 385 393 10.1111/cns.13258 31729196 PMC7052804

[45] Shih Y.T. Hsueh Y.P 2018 The involvement of endoplasmic reticulum formation and protein synthesis efficiency in VCP- and ATL1-related neurological disorders J. Biomed. Sci. 25 2 10.1186/s12929-017-0403-3 29310658 PMC5757295

[46] Reynisdottir I. Arason A. Freysteinsdottir E.S. Kristjansdottir S.B. Hilmarsdottir B. Traustadottir G.A. et al 2023 High Atlastin 2-2 (ATL2-2) Expression Associates with Worse Prognosis in Estrogen-Receptor-Positive Breast Cancer Genes (Basel) 14 1559 10.3390/genes14081559 37628611 PMC10454310

[47] Zhang M. Hu J 2013 Homotypic fusion of endoplasmic reticulum membranes in plant cells Front. Plant Sci. 4 514 10.3389/fpls.2013.00514 24385977 PMC3866526

[48] Yan L. Sun S. Wang W. Shi J. Hu X. Wang S. et al 2015 Structures of the yeast dynamin-like GTPase Sey1p provide insight into homotypic ER fusion J. Cell Biol. 210 961 972 10.1083/jcb.201502078 26370501 PMC4576867

[49] Anwar K. Klemm R.W. Condon A. Severin K.N. Zhang M. Ghirlando R. et al 2012 The dynamin-like GTPase Sey1p mediates homotypic ER fusion in S. cerevisiae J. Cell Biol. 197 209 217 10.1083/jcb.201111115 22508509 PMC3328390

[50] Krishna S. Ford M.G.J 2023 The atlastin paralogs: The complexity in the tails J. Cell Biol. 222 e202305116 10.1083/jcb.202305116 37327452 PMC10278267

[51] Jahn R. Scheller R.H 2006 SNAREs — engines for membrane fusion Nat. Rev. Mol. Cell Biol. 7 631 643 10.1038/nrm2002 16912714

[52] Cai H. Reinisch K. Ferro-Novick S 2007 Coats, Tethers, Rabs, and SNAREs Work Together to Mediate the Intracellular Destination of a Transport Vesicle Dev. Cell 12 671 682 10.1016/j.devcel.2007.04.005 17488620

[53] Gerondopoulos A. Bastos R.N. Yoshimura S.-I. Anderson R. Carpanini S. Aligianis I. et al 2014 Rab18 and a Rab18 GEF complex are required for normal ER structure J. Cell Biol. 205 707 720 10.1083/jcb.201403026 24891604 PMC4050724

[54] English A.R. Voeltz G.K 2013 Rab10 GTPase regulates ER dynamics and morphology Nat. Cell Biol. 15 169 178 10.1038/ncb2647 23263280 PMC3582403

[55] Miyazaki K. Wakana Y. Noda C. Arasaki K. Furuno A. Tagaya M 2012 Contribution of the long form of syntaxin 5 to the organization of the endoplasmic reticulum J. Cell. Sci. 125 5658 5666 10.1242/jcs.105304 23077182

[56] Zhang M. Wu F. Shi J. Zhu Y. Zhu Z. Gong Q. et al 2013 ROOT HAIR DEFECTIVE3 family of dynamin-like GTPases mediates homotypic endoplasmic reticulum fusion and is essential for Arabidopsis development Plant Physiol. 163 713 720 10.1104/pp.113.224501 23922269 PMC3793052

[57] Zheng H. Chen J 2011 Emerging aspects of ER organization in root hair tip growth: lessons from RHD3 and Atlastin Plant Signal. Behav. 6 1710 1713 10.4161/psb.6.11.17477 22057320 PMC3329342

[58] Wang N. Rapoport T.A 2019 Reconstituting the reticular ER network – mechanistic implications and open questions J. Cell. Sci. 132 10.1242/jcs.227611 30670475

[59] Hu J. Shibata Y. Voss C. Shemesh T. Li Z. Coughlin M. et al 2008 Membrane proteins of the endoplasmic reticulum induce high-curvature tubules Science 319 1247 1250 10.1126/science.1153634 18309084

[60] Voeltz G.K. Prinz W.A. Shibata Y. Rist J.M. Rapoport T.A 2006 A class of membrane proteins shaping the tubular endoplasmic reticulum Cell 124 573 586 10.1016/j.cell.2005.11.047 16469703

[61] Espadas J. Pendin D. Bocanegra R. Escalada A. Misticoni G. Trevisan T. et al 2019 Dynamic constriction and fission of endoplasmic reticulum membranes by reticulon Nat. Commun. 10 5327, 5327 10.1038/s41467-019-13327-7 31757972 PMC6876568

[62] Park S.H. Zhu P.-P. Parker R.L. Blackstone C 2010 Hereditary spastic paraplegia proteins REEP1, spastin, and atlastin-1 coordinate microtubule interactions with the tubular ER network J. Clin. Invest. 120 1097 1110 10.1172/JCI40979 20200447 PMC2846052

[63] Chen S. Novick P. Ferro-Novick S 2012 ER network formation requires a balance of the dynamin-like GTPase Sey1p and the Lunapark family member Lnp1p Nat. Cell Biol. 14 707 716 10.1038/ncb2523 22729086 PMC3389217

[64] Chen S. Desai T. McNew J.A. Gerard P. Novick P.J. Ferro-Novick S 2015 Lunapark stabilizes nascent three-way junctions in the endoplasmic reticulum Proc. Natl. Acad. Sci. U.S.A. 112 418 423 10.1073/pnas.1423026112 25548161 PMC4299238

[65] Sun J. Movahed N. Zheng H 2020 LUNAPARK Is an E3 Ligase that mediates degradation of ROOT HAIR DEFECTIVE3 to maintain a tubular ER network in arabidopsis Plant Cell 32 2964 2978 10.1105/tpc.18.00937 32616662 PMC7474291

[66] Anggrandariyanny P.C. Kajiho H. Yamamoto Y. Sakisaka T 2022 Lunapark ubiquitinates atlastin-2 for the tubular network formation of the endoplasmic reticulum J. Biochem. 172 245 257 10.1093/jb/mvac060 35894092

[67] Jang E. Lee M. Yoon S.Y. Lee S.S. Park J. Jin M.S. et al 2023 Yeast lunapark regulates the formation of *trans*-Sey1p complexes for homotypic ER membrane fusion iScience 26 108386 10.1016/j.isci.2023.108386 38025788 PMC10679814

